# The impact of hydropower dam construction on malaria incidence: Space-time analysis in the Brazilian Amazon

**DOI:** 10.1371/journal.pgph.0001683

**Published:** 2023-03-20

**Authors:** Igor C. Johansen, Emilio F. Moran, Marcelo U. Ferreira

**Affiliations:** 1 Center for Environmental Studies and Research–Nepam, State University of Campinas, Campinas, SP, Brazil; 2 Center for Global Change and Earth Observations and Dept. of Geography, Environment and Spatial Sciences–Michigan State University, East Lansing, MI, United States of America; 3 Department of Parasitology, Institute of Biomedical Sciences–University of São Paulo, São Paulo, SP, Brazil; 4 Global Health and Tropical Medicine, Institute of Hygiene and Tropical Medicine, Nova University of Lisbon, Lisbon, Portugal; Nepal Health Research Council, NEPAL

## Abstract

During the first two decades of the 21st century, Brazil carried out massive public investments on infrastructure projects, such as large hydropower dams, with potential impact on population health. Here we characterize local malaria transmission and its potential spread during the construction of three large hydropower dams in the Brazilian Amazon. We focus on Porto Velho (PVH), in Rondônia state, where the Santo Antônio and Jirau dams were built (2008–2013), and Altamira region (ATM), in Pará state, where the construction of the Belo Monte dam took place (2011–2016). Analyzed data cover 4 years before, 6 years during, and 4 years after each dam construction. In total, we utilized malaria case notifications entered into the electronic malaria notification system of the Ministry of Health of Brazil between January 2004 and December 2020 (n = 39,977,167 malaria notifications). First, we used Interrupted Time-Series Analysis (ITSA) to assess temporal changes in malaria notifications in the study sites. Then, we conducted a space-time cluster analysis to investigate the potential of malaria spread from the study sites (sources) to elsewhere (sinks). Finally, we present the sociodemographic characteristics of exported cases over time using multivariate logistic regressions. Our results show that there was no upsurge in malaria cases in the study sites and exported cases did not trigger outbreaks in other localities. Exported malaria infections originating from PVH and ATM were typically found in working age literate males involved in mining, farming or traveling. We suggest that efficient control measures, such as ensuring timely diagnosis and treatment; fostering integrated vector control; promoting health education; and prevention, detection and containment of outbreaks, if properly implemented and sustained, may prevent local and introduced malaria outbreaks during and after hydropower dam construction in the Amazon.

## Introduction

During the first two decades of the 21^st^ century, Brazil experienced an economic boom that led the Federal Government to invest in large infrastructure projects countrywide. One of the main sectors receiving massive investments was the construction of large hydropower dams to assure energy self-sufficiency and sustain the economic growth in the country in the long term [1]. This was part of a national strategy to create jobs, through the Program of Accelerated Growth (Portuguese acronym, PAC), to mitigate the impact of the global recession of 2008 [2].

Since the most populated regions in Brazil are the Southeast, Northeast and South–with respectively 42%, 27% and 14% of the country’s population [3]–, that do not have much hydropower potential remaining, the Amazon is the new frontier explored for dam construction [4]. This frontier also comprises the highest concentration of drinkable water in the world, most of it in rushing rivers [5]. Three of the main dams that took place in this context were Santo Antônio and Jirau, built between 2008 and 2013 on the Madeira Basin, in Rondônia state; and Belo Monte, built between 2011 and 2016 on the Xingu Basin, in Pará state. The three are considered large dams, with installed capacity of 3 GW for Santo Antônio, 3 GW for Jirau, and 11 GW for Belo Monte [6].

*Anopheles darlingi* mosquitos, the main malaria vector in the Brazilian Amazon [7, 8], use as a main ecological habitat shaded stagnant water, preferably close to dense vegetation. As a consequence, currently 99% of the malaria cases in Brazil occur in the Amazonian region [9, 10], where these large infrastructure projects have been built.

Hydropower in Brazil is mostly based on dams, which, and despite technology advancements, result in extensive flooded areas [11]. In the Amazon, the environmental impact of dams is aggravated by the flat topography, increasing further the flooded surface area. Previous studies have shown that malaria can be triggered by hydropower dams construction in multiple ways: stagnant water areas in the dam reservoir may increase the abundance of *Anopheles* mosquitoes; building dams puts humans and infected malaria vectors in close contact, as shown in an extensive literature about malaria and frontier expansion in the Brazilian Amazon [9, 12, 13]; the arrival of a large influx of population, which can be infected with malaria parasites, can “import” malaria cases and propel the availability of the pathogens for local vectors [14]; and this population mobility can “export” malaria cases to other localities since this workforce has a high turnover.

Hydropower dam construction in Brazil requires Environmental Impact Assessments and Environmental Impact Reports (Portuguese acronym, EIA-RIMAs) that evaluate the environmental impact of the project, considering socio-economic, cultural and human-health aspects. Mitigation strategies must be proposed [15–17]. If the actions are not properly addressed, dam construction or operation licenses may be revoked. The plan has to incorporate public health considerations. Indeed, malaria was recognized as endemic in the regions by the EIA-RIMAs of both study sites, and the reports pointed out strategies to cope with the disease during the construction to avoid an upsurge [18, 19].

We focus on malaria since it is the most persistent endemic disease in the region [8]. Investigations prior to the dam’s construction in Porto Velho municipality (PVH), in Rondônia state, where Santo Antônio and Jirau dams were built (2008–2013), pointed out to the high risk of a malaria upsurge in the area due to the favorable socio-environmental and epidemiologic local conditions [20, 21]. However, there are no studies focused on what happened to malaria notifications during the entire period of construction, but only for a part of that period [22].

In contrast, in another setting, Altamira region (ATM), in Pará state, where Belo Monte dam construction (2011–2016) took place, a research investigated the malaria notifications only during the construction and found a clear fall in the occurrences of the disease [23], but it did not reach the moment after the end of the construction, when control efforts can fade.

Besides, most analyses of the relation between hydropower dams construction and malaria occurrences focus on cases transmitted locally and notified in the same location [14, 24, 25] or are based on narrow time frames [22, 23].

To fill these gaps in the literature our paper aims to investigate the following research questions and hypotheses by comparing the two study settings (PVH and ATM) over a period of analysis of 4 years before, 6 years during and 4 years after each dam’s construction (14 years total):

First, we want to investigate whether the fall in malaria notifications observed in ATM [23] also occurred in PVH and if this fall is maintained after the end of the construction. Since the projects are contemporary, the regulatory measures are the same and so should be the results in terms of malaria control. We expect an increase after the end of the construction, when control efforts may be reduced.

Second, regarding the exported cases (infections occurring in the study settings, but notified elsewhere), we wanted to check if they have potential to spread malaria to other localities and if we find differences among the study areas. Our hypothesis is that, since locally transmitted cases have been reduced, we expect the same for exported cases, reducing the potential of spread. However, there are differences. Considering that Porto Velho is a capital with more population and more connected to the country via roads and well-structured airport, we should see a higher potential of this area to produce more exported cases to further distances. We also want to know what are the sociodemographic characteristics of these exported cases. According to previous studies, we expect to find most cases among males of working age [21, 26, 27].

This study leverages a rich malaria dataset to present the impact of these infrastructure projects in malaria notifications. We aim to provide new insights for malaria control and elimination in the Brazilian Amazon. Additionally, this analysis fosters advances in knowledge in light of current economic development policies that are based on building large infrastructure projects, which is a trend in many countries of the Global South [28, 29].

## Methods

### Study sites

**Fig 1:**
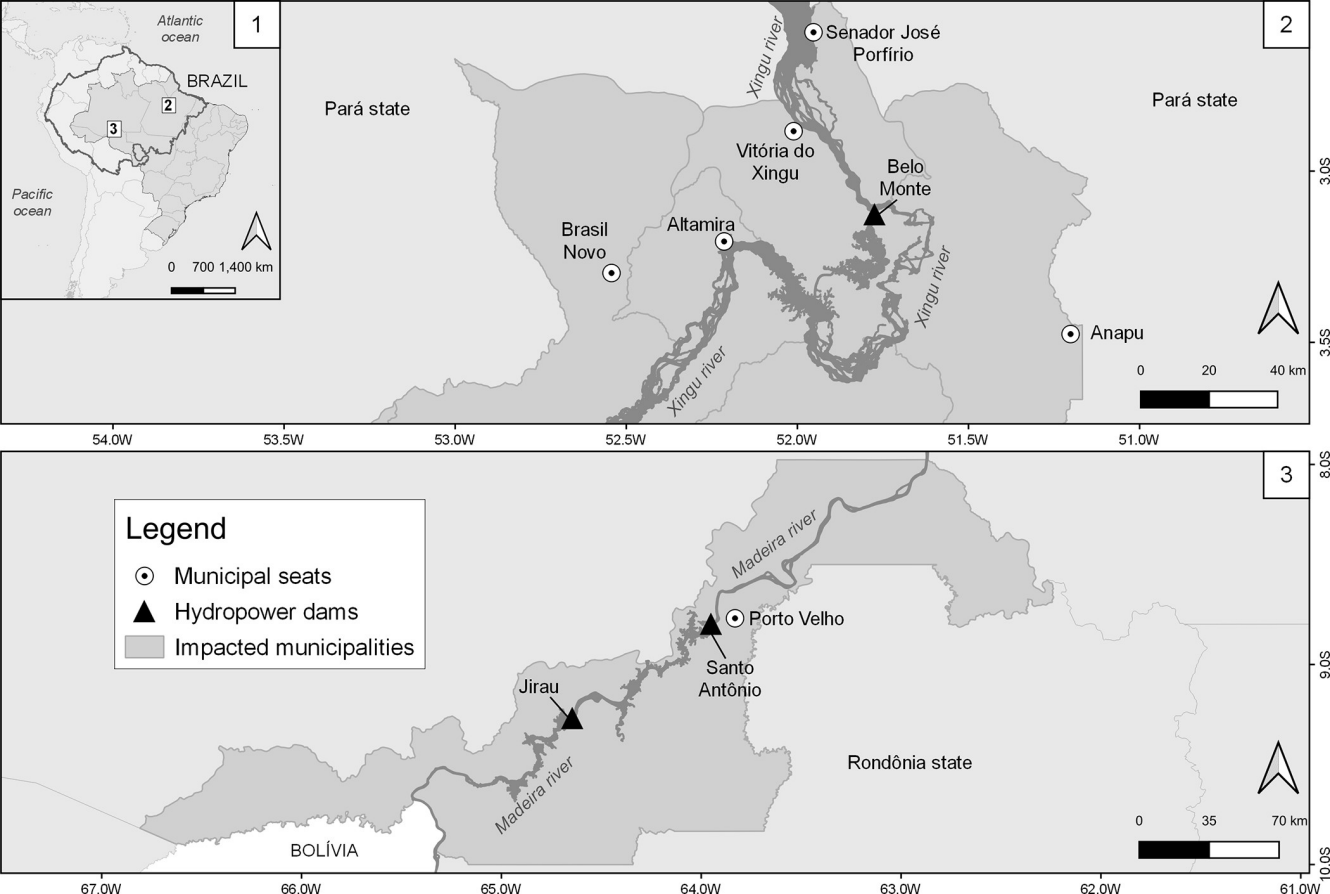
Location of the study sites, the municipalities of Porto Velho, in the Madeira river basin, Rondônia state; and Altamira, Anapu, Brasil Novo, Senador José Porfírio and Vitória do Xingu, in the Xingu river basin, Pará state, Brazil. 1: South America, Brazilian Federal Units and the Amazon basin (continuous line crossing international borders); 2: Altamira and neighboring municipalities (ATM) impacted by Belo Monte dam; 3: Porto Velho municipality (PVH), impacted by Santo Antônio and Jirau dams. Darker gray areas represent the municipalities impacted by dams’ construction. The triangles show the dams’ location, and the darkest grey thick lines represent the rivers. Municipal seats are represented with white circles. Figure created with QGIS software version 3.14, an open source Geographic Information System (GIS) licensed under the GNU General Public License (https://bit.ly/2BSPB2F). Base layer sources: South America shape file retrieved from the Database of Global Administrative Areas (GADM) website (https://bit.ly/3ZqwJ4u), under an open license (CC-BY): http://bit.ly/3Zl2rjE; shape file representing the Brazilian administrative areas provided from the Brazilian Institute of Geography and Statistics (IBGE) website (https://bit.ly/3vWaZjC), under an open license (CC-BY): https://bit.ly/3W2qxNk.

The current study is part of a broader, long-term research project into the effects of hydropower projects in multiple river basins in the Brazilian Amazon. This project comprises regional partnerships with universities, surveys, qualitative interviews, qualitative field observations, and document analysis. For this paper, we rely upon secondary data provided by the Ministry of Health of Brazil. The study sites are the municipalities of Porto Velho, in the Madeira river basin, and Altamira, Anapu, Brasil Novo, Senador José Porfírio and Vitória do Xingu, in the Xingu river basin. They cover a surface area of 34,090km^2^ in the westernmost Amazon and 195,300km^2^ in the eastern Brazilian Amazon, respectively (Fig 1). Porto Velho is also the capital of Rondônia state, presenting a total population of 548,952 inhabitants in 2021, while the study site in Pará state comprise the following population: Altamira (117,320); Anapu (29,312); Brasil Novo (14,883); Senador José Porfírio (11,305); and Vitória do Xingu (15,421), a total of 188,241 inhabitants [3].

### Data

We retrieved all malaria case notifications from the Malaria Epidemiological Surveillance Information System (SIVEP) of the Ministry of Health of Brazil between January 2004 and December 2020 (*n* = 39,977,167 notifications, see the flowchart in S1 Fig). Because malaria is a notifiable disease in Brazil and diagnostic testing and treatment are not available outside the network of government-run health care facilities, the database comprises the vast majority of laboratory-confirmed malaria episodes countrywide [30].

**Fig 2:**
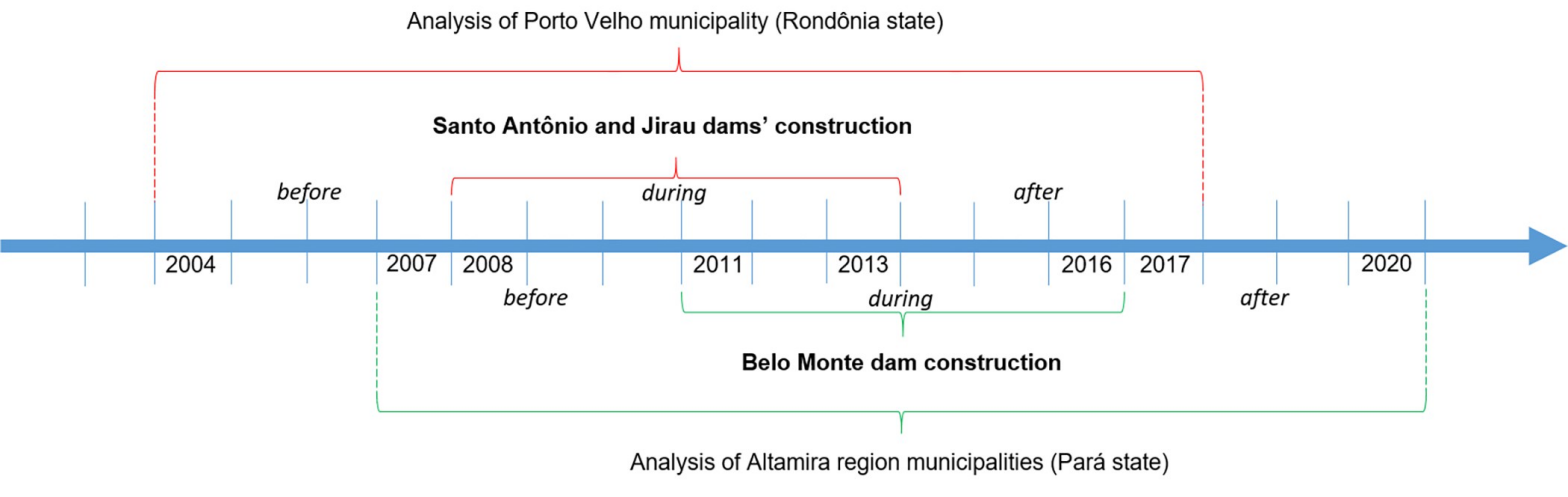
Time scale for the analysis of malaria in Velho municipality (PVH) and Altamira region municipalities (ATM), 2004–2017 and 2007–2020, respectively. Source: Elaborated by the authors.

From the electronic malaria notification system, we created two datasets: one for PVH municipality and another for ATM region. The difference in the number of municipalities studied in each area is a result of the fact that, in the case of Santo Antônio and Jirau dams, the only municipality directly affected was Porto Velho, while for Belo Monte construction 5 municipalities were impacted, i.e., Altamira, Anapu, Brasil Novo, Senador José Porfírio and Vitória do Xingu [18, 19]. The period of analysis for each dataset comprised 4 years before, 6 years during and 4 years after the dams’ construction. Santo Antônio and Jirau were built simultaneously, between 2008 and 2013, while Belo Monte was constructed between 2011 and 2016 (Fig 2).

### Research methods

Our research methods are aligned with the research questions as follows:

We used Interrupted Time-Series Analysis (ITSA) to investigate whether the fall in malaria notifications observed in ATM [23] also occurred in PVH and if this fall is maintained after the end of the construction. We used all municipalities from the Brazilian Amazon as a comparator (controls) and defined as the “time of intervention” two moments that affect malaria incidence: the start and the end of the hydropower construction in each study site. This strategy allows us to investigate if temporal changes in malaria notifications in each study site followed the overall trend for the Amazon region as a whole, at a significance level of 0.05 [31].

To check if exported cases have potential to spread malaria to other localities and if we find differences among the study areas, we conducted a space-time cluster analysis using the software SaTScan v 10.0.1 [32]. We utilized a Space-Time Permutation Probability Model with the parameters: minimum temporal cluster size of 1 year and maximum temporal cluster size of 15 percent of the study period. This model requires only case data, with information about spatial location and time (year) for each exported case. There is a spatial cluster if, during a specific time period, the study site (covering some municipalities) presents a higher proportion of cases compared to the other geographical areas. Only statistically significant space-time clusters are presented in the results, with a threshold of p<0.01. Maps were drawn with QGIS software version 3.14.

To analyze the sociodemographic characteristics of the exported cases we first summarized them based on the notification system variables. Pearson’s Chi-Square (χ^2^) test of differences was applied to assess sociodemographic and epidemiologic characteristics of the exported cases before, during and after the dams’ construction in each study site. Temporal trends were described from 2004 to 2017 for PVH and from 2007 to 2020 for ATM (Fig 2). Our analysis evaluated space and time malaria dynamics, including the following explanatory variables: sex (female, male); age group (<5 years old, 5–15, 16–24, 25–40, 41–64, and over 65); symptoms (asymptomatic, symptomatic); occupation (other, agriculture, domestic, forestry, hunter/fisherman, miner, traveling, road/dam builder); species (mixed/other; *Plasmodium vivax*, *Plasmodium falciparum*), schooling (illiterate, elementary school, high school, college); mean, median, minimum, maximum and standard deviation distances from municipality of malaria occurrence to municipality of notification (in kilometers); and mean, median, minimum, maximum, and standard deviation of patients’ age (in years). Then, we ran logistic regressions for the analysis of epidemiological and sociodemographic characteristics of exported cases. Only variables that reached the statistical significance of P<0.001 in the unadjusted models (univariate regressions) were considered for the adjusted models (multivariate regressions). Data analysis was conducted in R version 3.4.2 [33]. Trends were assessed using the dplyr package, and distances were calculated using geodist package, tools that are freely available through the software R.

### Ethics

This study was approved by the Institutional Review Board of the State University of Campinas (protocol number: 51839921.8.0000.8142).

## Results

The dataset to investigate malaria dynamics over time in both study sites comprised 39,977,167 malaria case notifications, of which 313,905 were eligible for the study of PVH and 36,066 for investigating ATM. From the total eligible malaria notifications, in PVH 232,087 were locally transmitted malaria cases; 42,659 were imported to and 39,159 were exported from the municipality between 2004 and 2017. In ATM, 28,272 locally transmitted malaria cases were notified; 4,778 were imported to and 3,016 were exported from the municipalities in the region (S1 Fig and S1 Table).

**Fig 3:**
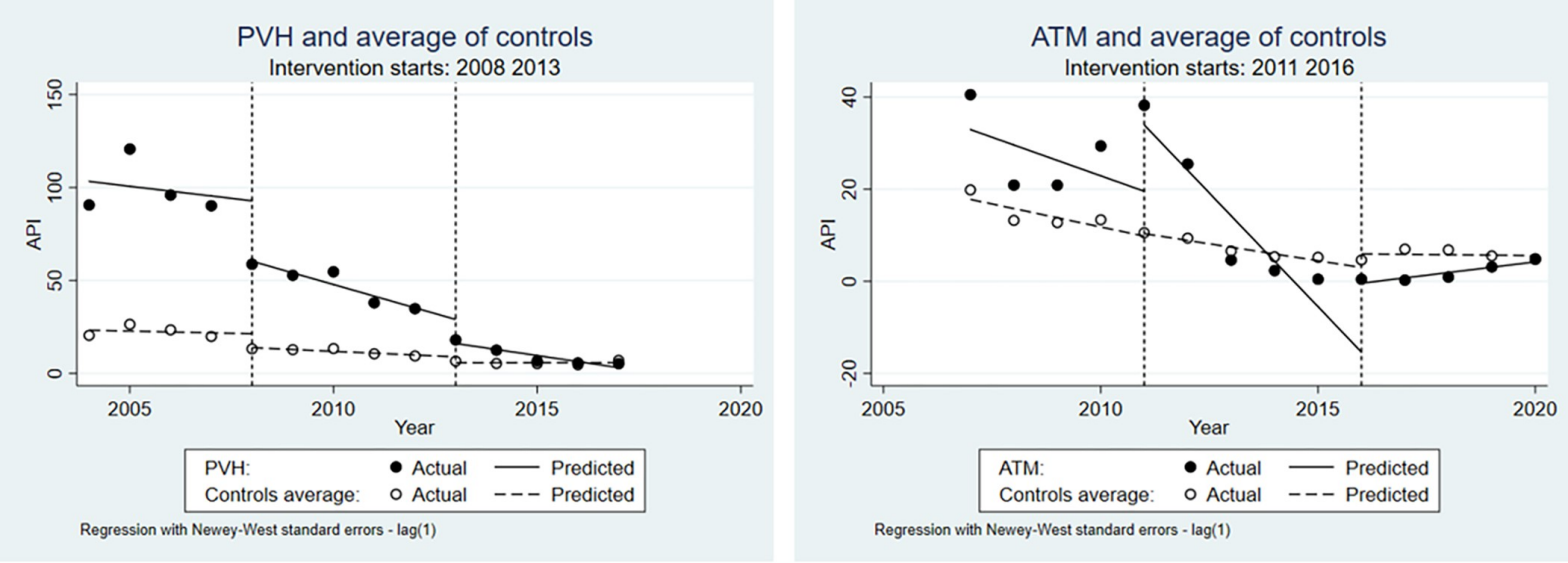
Interrupted time-series regression analysis of malaria annual parasite index (cases per 1,000 inhabitants) in each study site, PVH and ATM (black dots and solid lines) and Amazon as control (open dots and dashed lines) before, during and after the dams’ construction. The vertical dashed line separates pre- and post- “intervention”, in this case the start and end of the dam’s construction. The construction in PVH starts in 2008 and ends in 2013, while in ATM it starts in 2011 and ends in 2016.

Malaria incidence declined during the dam’s construction period in both study sites (Fig 3). We found a statistically significant trend comparing the time before, during and after dam construction. The difference between level and slope of the study cases compared to the Amazon as control region is also significant, especially before and during the construction. For the moment before the construction, we notice higher levels of malaria incidence in both study sites, although in PVH it is much higher than in the Amazon. During the construction, there is a clear reduction in the incidence, even more noticeable for ATM, reaching levels close to zero. In the post-construction moment, both study sites tend to converge to malaria incidence levels observed in the Amazon as a whole.

**Fig 4:**
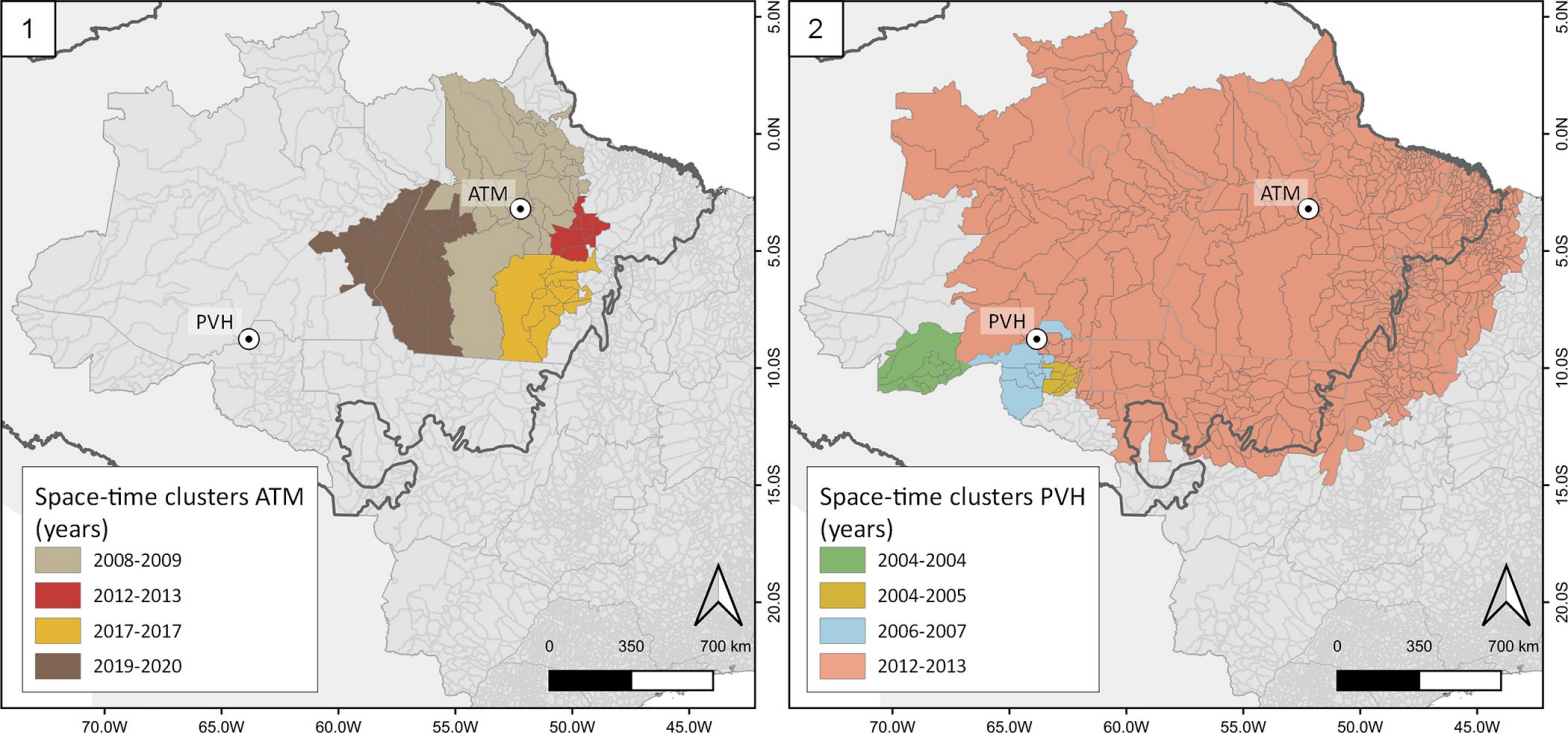
Space-time clusters of malaria exported cases from the study areas. 1. Clusters of malaria exported cases from Altamira region (ATM), by year, for the period 2007 to 2020. 2. Clusters of malaria exported cases from Porto Velho municipality (PVH), by year, for the period 2004 to 2017. The thick grey line represents the Amazon basin. The spatial units are Brazilian municipalities, represented as light gray areas. Arrows indicate solely the direction of the malaria cases flow, from the sources (ATM and PVH) to the sinks (other areas in the country). Figure created with QGIS software version 3.14, an open source Geographic Information System (GIS) licensed under the GNU General Public License (https://bit.ly/2BSPB2F). Base layer sources: South America shape file retrieved from the Database of Global Administrative Areas (GADM) website (https://bit.ly/3ZqwJ4u), under an open license (CC-BY): http://bit.ly/3Zl2rjE; shape file representing the Brazilian administrative areas provided from the Brazilian Institute of Geography and Statistics (IBGE) website (https://bit.ly/3vWaZjC), under an open license (CC-BY): https://bit.ly/3W2qxNk.

To investigate whether exported cases have potential to spread malaria to other localities and if we find differences among the study areas, we begin by analyzing the spatial-temporal dynamics of exported cases from ATM and PVH. We show that that the statistically significant clusters of municipalities receiving exported malaria cases from ATM tended to increase from neighboring areas in the period before the construction (2007 to 2010) to more distant areas in the following years (Fig 4, panel 1). The same occurs in PVH, which presented clusters of municipalities nearby receiving most malaria exported cases before the dam construction (2004–2007), but then reaching more distant areas (Fig 4, panel 2).

PVH not only presented a higher capability of reaching further areas but also with more exported cases, as presented in the S1 Table. PVH showed more than 10 times the total exported malaria cases compared to ATM during the same interval of 14 years of analysis. However, when looking at the API for all the 20 main sinks of malaria cases from the study sites, none of them presented outbreaks in the period of analysis, by the contrary, these sinks followed the same decreasing pattern in malaria notifications observed in the Amazon as a whole.

Data presented in S2 Table details the sociodemographic characteristics of the exported cases. In both localities, around 3 out of 4 exported malaria infections were found among males; close to 80% of them were in individuals aged 16 to 64 years old; the vast majority being symptomatic *P*. *vivax* cases; and most have elementary school (complete or incomplete) as the highest schooling degree.

In ATM, work status of exported malaria cases before construction was based mainly on agriculture and hunter/fisherman, but during construction miner was the most relevant economic activity of exported cases. In PVH, agriculture was the occupation of most exported cases before, during and after dams’ construction. In both study sites the proportions of occupation marked as “other” and missing was high, while the category road/dam builder presented only residual importance.

In ATM and PVH max distances between sources and sinks occurred during the dams’ construction. Standard deviation of this distance was higher for PVH (355.5 km) compared to ATM (276.5 km) during the dams’ construction.

S3 Table shows the characteristics of malaria infections whose sources were ATM or PVH during the entire study period, while S4 and S5 Tables show results, respectively, for ATM or PVH, separated by the periods before, during and after dams’ construction. Exported infections present a predominance of men compared to women (S3 Table), this trend was consistent inclusive when analyzed by period (S4 and S5 Tables). The 16+ years old population is the preferential focus of malaria exported cases to other areas.

Results were not consistent about presence of symptoms among exported cases. In ATM and PVH, miners and travelers present the highest likelihood of being an exported malaria case compared to other occupations, and in PVH those who work on agriculture are also important carriers of this disease to other locations. Dam builders are not statistically significant in none of the study areas (S3 Table). It is noticeable that in ATM miners presented the highest likelihood of being an exported case during the dam construction (with odds more than 19 times higher than other occupation) (S4 Table), while in PVH travelers were the most important carriers of malaria to elsewhere, independently of the moment of the construction (S5 Table).

Exported cases from ATM and PVH were mostly literate, presenting at least complete or incomplete elementary school, what was consistent for both study areas and across the different moments analyzed (S3–S5 Tables).

## Discussion and conclusion

We have shown that hydropower dam construction in ATM and PVH, between 2008 and 2016, has not been associated with increased local malaria transmission or malaria outbreaks elsewhere in the Brazilian Amazon. These findings contrast with the well-established link between human mobility and malaria that characterized the entire process of occupation of the Amazon, especially in the second half of the 20^th^ century. Human mobility spreads malaria parasites to move over extended areas and expose malaria-naïve populations, who did not know this disease in their places of origin, to increased risk of infection and severe disease [9, 34, 35]. We argue that, because malaria control was perceived as a top priority, efficient control measures were implemented during dam construction in ATM and PVH [18, 19, 36]. Nevertheless, a range of health issues were less successfully addressed in construction sites, such as syphilis in pregnant women, violence-related deaths (homicides) and traffic accidents [37, 38]. In other words, malaria is an exception to a norm concerning the health impacts of large hydropower dams in the Brazilian Amazon.

Besides, there are numerous other socio-environmental damaging consequences of those large infrastructure projects in the region. Examples are losses in social capital and decreasing self-rated health [39]; increasing injustices to the local populations for not being properly compensated by their losses [40]; questionable processes of consultation of the host population, lacking effective participation [41]; displacement of thousands of people, tending to impact marginalized populations more intensely [29, 42]; and environmental damage to river ecosystems and to fish biodiversity [43, 44].

While previous studies investigated a variety of impacts of hydropower dams on local populations’ health [7, 22, 23, 25, 27, 39, 45, 46], to the best of our knowledge none of them have analyzed locally acquired and exported malaria infections over an extended time frame, covering the periods before, during and after the construction. The present investigation aimed to fill this gap. By investigating whether the fall in malaria notifications observed in ATM [23] also occurred in PVH and if this fall is maintained after the end of the construction, we confirmed our hypothesis that during construction there was no escalation in malaria incidence in PVH and ATM, but we were somewhat surprised by the fact that malaria cases continue at low levels even after the end of construction. Possibly, a longer post-construction period of analysis will be needed to assess if malaria notifications will be maintained at low levels. The fall in malaria notifications is consistent during the period of analysis of 14 years, despite the massive environmental changes, the greater population mobility to, from and within the study areas and the dramatic increase in the population [25, 27]. Yet, it is important to note that in this period the Brazilian Amazon as a whole also consistently presented decreasing malaria figures [47].

To answer the question if exported cases have potential to spread malaria to other localities and if we find differences among study areas, results showed that exported cases fell as well as locally transmitted malaria cases, reducing the potential of the spread from the study sites do other areas. However, confirming our hypothesis, PVH emerged as a more important source of exported malaria infections compared with ATM. This finding can be linked to the facts that: 1) PVH presents higher population than ATM, consequently more people exposed to malaria infections and to travel to other regions; 2) PVH is a capital, well provided with terrestrial and aerial infrastructure for population mobility, while ATM is more difficult access; 3) dams’ construction in PVH occurred before ATM, allowing the population movement from one construction site to the other, in the opposite side of the Amazon region; and 4) after Belo Monte construction in ATM there were no large infrastructure projects with the same scale in the Amazon anymore, which can have contributed to reduced population mobility and more locally-restricted exported malaria cases from ATM. The absence of large infrastructure projects after Belo Monte in ATM is related to political and economic crises that the country faced after 2014 [48]. The analysis of sources and sinks of malaria cases is evolving and puts light on the dynamic processes of malaria mobility through human beings across space and over time [49–51], but this was not the case for our study areas.

Regarding the analysis of the sociodemographic characteristics of the exported cases, this paper showed that they were, in both study sites: males; 16+ years old; literate; and whose economic activities are related to mining, agriculture or traveling (S3–S5 Tables). For understanding travelers as the ones with the highest likelihood of being an exported malaria case we should analyze the way this information is collected in the notification form. Actually, it is asked the activity that most probably was the cause of the malaria infection, to allow the health system to evaluate risk activities. Since we are focusing on exported cases, all of them were, to some extent, travelers. Thus, this information does not accurately capture the nature of the activity that triggered malaria infection. Even so, in this study the occupation data highlighted other activities relevant for malaria occurrence, as is the case of agriculture and mining, two outdoor activities that can increase exposure to malaria vector. Dam builders were less relevant than we would expect, but this finding is in line with the results previously shown. The results suggest that the sociodemographic profile of exported cases found in this study is not very different from the regular profile of an infected person with malaria in the Brazilian Amazon [21, 26, 27].

This study has some limitations. First, we rely on secondary data to assess the temporal and spatial patterns of malaria occurrence in the study settings. Although the information utilized presents gaps depending on the variable of interest, this is still the most comprehensive data on malaria available in Brazil, since malaria notifications are mandatory in the country. Second, and related to that, the large proportion of “other” among the occupation categories suggests that this variable should be analyzed with caution.

This paper suggests that, while there are a range of well-known adverse social, environmental and economic consequences of dam construction in the Amazon [29, 37, 38, 40, 41, 43, 44, 52, 53], malaria control appears to have succeeded in our study sites. However, once malaria incidence is reduced as does the funding for surveillance and control, transmission could rebound quickly. Therefore, we advocate for the maintenance for the long term the structure made available for malaria control in both study areas and future dams.

Finally, we highlight two key aspects to put those results in perspective. The first is that malaria control by dam builders is not a gift. It is mandatory, assured by the Resolution 286 of the Brazilian National Environment Council (Portuguese acronym, CONAMA), which obliges dam builders to develop and implement a malaria control program for endemic areas that will receive these projects in order to get approval for their environmental licensing [17]. Second, the basis of the successful programs conducted in both study sites are: ensuring timely diagnosis and treatment; fostering integrated vector control; promoting health education; and prevention, detection and containment of outbreaks [23]. These are required in the National Malaria Control Program [54]. We have provided proof that these measures work. Now we need the same level of funding to put it in practice in all of the remaining malaria endemic localities of the Brazilian Amazon.

## Supporting information

S1 Checklist Strobe checklist. (DOC)

S1 Fig Flowchart indicating the data filtering process to select the eligible malaria cases for the study of malaria in Porto Velho (PVH) municipality and in Altamira region municipalities (ATM). (TIF)

S1 Table Malaria cases by type (locally transmitted, imported and exported), for Porto Velho municipality (PVH) and Altamira region municipalities (ATM), 2004–2017 and 2007–2020 respectively. (DOCX)

S2 Table Descriptive statistics of malaria exported cases, from Altamira region (ATM) or Porto Velho municipality (PVH), before, during and after dams’ construction. (DOCX)

S3 Table Correlates of malaria infections whose sources were Altamira region (ATM) or Porto Velho municipality (PVH), 2004–2017 and 2007–2020 respectively. (DOCX)

S4 Table Correlates of malaria infections whose sources was Altamira region (ATM)–before (2007–2010), during (2011–2016) and after dam’s construction (2017–2020). (DOCX)

S5 Table Correlates of malaria infections whose sources was Porto Velho municipality (PVH)–before (2004–2007), during (2008–2013) and after dam’s construction (2014–2017). (DOCX)
