# Supplementary figures and images for: The impact of hydropower dam construction on malaria incidence: Space-time analysis in the Brazilian Amazon
Source: PLOS Glob Public Health. 2023 Mar 20;3(3):e0001683. doi: 10.1371/journal.pgph.0001683 (PMC10027221; doi:10.1371/journal.pgph.0001683)

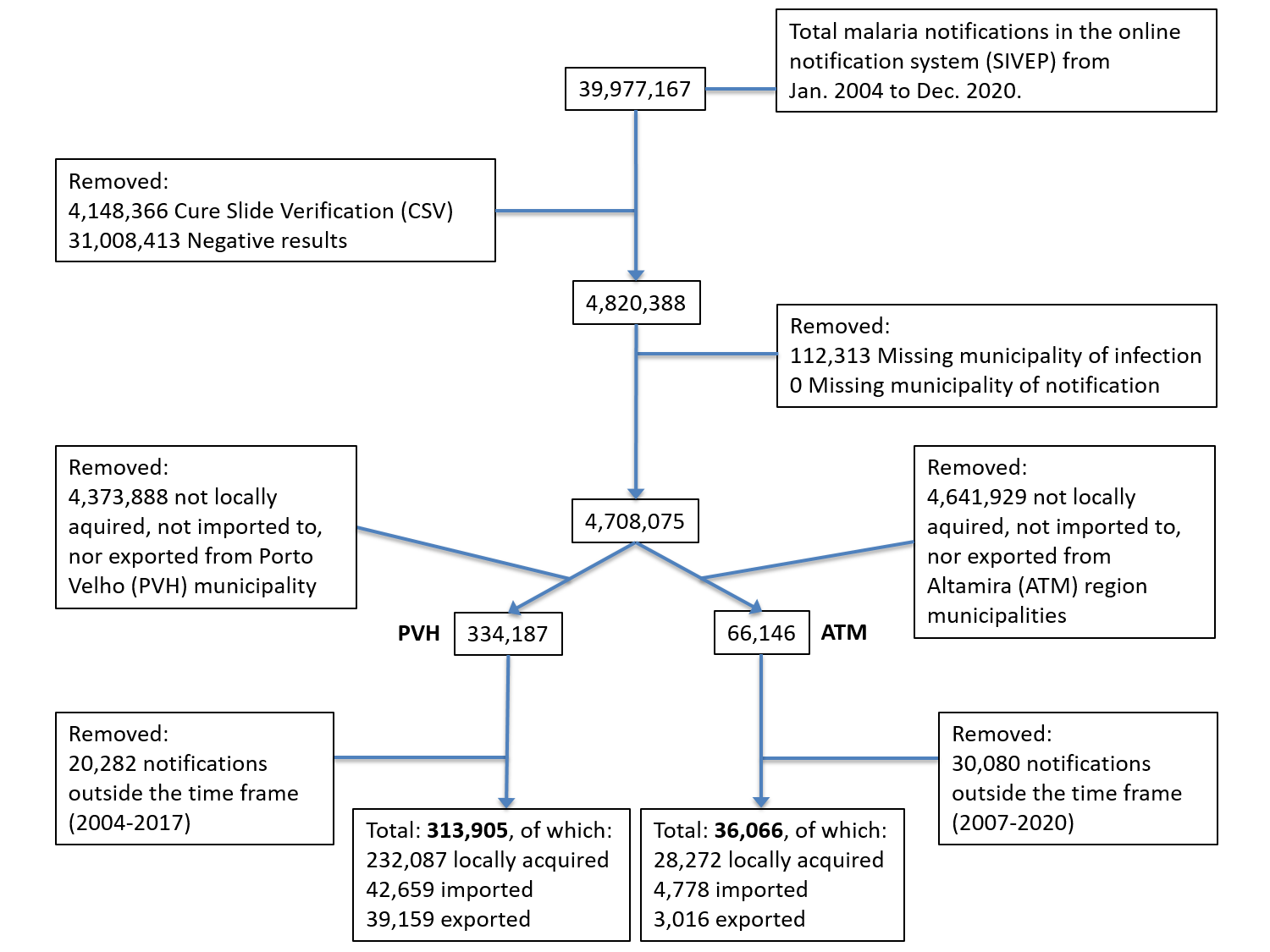

Supplement: S1 Fig — (TIF) [file pgph.0001683.s002.tif]
