# Supplementary material for: The impact of hydropower dam construction on malaria incidence: Space-time analysis in the Brazilian Amazon
Source: PLOS Glob Public Health. 2023 Mar 20;3(3):e0001683. doi: 10.1371/journal.pgph.0001683 (PMC10027221; doi:10.1371/journal.pgph.0001683)
Supplement: S1 Table — (DOCX) [file pgph.0001683.s003.docx]

**S1 Table.** Malaria cases by type (locally transmitted, imported and exported), for Porto Velho municipality (PVH) and Altamira region municipalities (ATM), 2004-2017 and 2007-2020 respectively

| PVH | | | | | ATM | | | | |
| --- | --- | --- | --- | --- | --- | --- | --- | --- | --- |
| Year | Time* | Locally transmitted | Imported | Exported | Year | Time* | Locally transmitted | Imported | Exported |
| 2004 | Before | 28,105 | 6,773 | 6,407 | 2007 | Before | 5,867 | 535 | 321 |
| 2005 | Before | 39,461 | 6,708 | 5,679 | 2008 | Before | 3,057 | 425 | 288 |
| 2006 | Before | 28,614 | 6,104 | 7,937 | 2009 | Before | 3,028 | 400 | 376 |
| 2007 | Before | 27,440 | 5,397 | 5,872 | 2010 | Before | 4,286 | 1,127 | 467 |
| 2008 | During | 19,761 | 3,815 | 2,512 | 2011 | During | 5,824 | 1,174 | 451 |
| 2009 | During | 18,285 | 2,242 | 1,931 | 2012 | During | 3,806 | 591 | 499 |
| 2010 | During | 20,907 | 2,235 | 2,526 | 2013 | During | 582 | 198 | 202 |
| 2011 | During | 14,514 | 2,678 | 2,035 | 2014 | During | 272 | 83 | 130 |
| 2012 | During | 13,859 | 1,999 | 1,562 | 2015 | During | 50 | 15 | 29 |
| 2013 | During | 7,837 | 1,230 | 901 | 2016 | During | 50 | 15 | 27 |
| 2014 | After | 5,539 | 1,038 | 624 | 2017 | After | 8 | 45 | 37 |
| 2015 | After | 2,965 | 604 | 398 | 2018 | After | 105 | 19 | 61 |
| 2016 | After | 2,425 | 664 | 446 | 2019 | After | 491 | 80 | 85 |
| 2017 | After | 2,375 | 1,172 | 329 | 2020 | After | 846 | 71 | 43 |
| Total |  | 232,087 | 42,659 | 39,159 | Total |  | 28,272 | 4,778 | 3,016 |

* Time according to dams’ construction in each locality.
