# Supplementary material for: The impact of hydropower dam construction on malaria incidence: Space-time analysis in the Brazilian Amazon
Source: PLOS Glob Public Health. 2023 Mar 20;3(3):e0001683. doi: 10.1371/journal.pgph.0001683 (PMC10027221; doi:10.1371/journal.pgph.0001683)
Supplement: S2 Table — (DOCX) [file pgph.0001683.s004.docx]

**S2 Table.** Descriptive statistics of malaria exported cases, from Altamira region (ATM) or Porto Velho municipality (PVH), before, during and after dams’ construction

|  |  | ATM | | | | | | PVH | | | | | |
| --- | --- | --- | --- | --- | --- | --- | --- | --- | --- | --- | --- | --- | --- |
|  |  | n | | | % | | | n | | | % | | |
|  |  | before | during | after | before | during | after | before | during | after | before | during | after |
| Gender | Female | 331 | 324 | 71 | 22.8 | 24.2 | 31.4 | 6,789 | 3,150 | 517 | 26.2 | 27.5 | 28.8 |
|  | Male | 1,121 | 1,014 | 155 | 77.2 | 75.8 | 68.6 | 19,106 | 8,317 | 1,280 | 73.8 | 72.5 | 71.2 |
|  | Total | 1,452 | 1,338 | 226 | 100.0 | 100.0 | 100.0 | 25,895 | 11,467 | 1,797 | 100.0 | 100.0 | 100.0 |
|  |  | χ² p-value<0.05 | | |  |  |  | χ² p-value<0.05 | | |  |  |  |
| Age group | < 5 years | 90 | 52 | 14 | 6.2 | 3.9 | 6.2 | 1,223 | 453 | 48 | 4.7 | 4.0 | 2.7 |
|  | 5 to 15 | 210 | 131 | 46 | 14.5 | 9.8 | 20.4 | 3,610 | 1,225 | 214 | 13.9 | 10.7 | 11.9 |
|  | 16 to 24 | 270 | 275 | 56 | 18.6 | 20.6 | 24.8 | 6,067 | 2,475 | 358 | 23.4 | 21.6 | 19.9 |
|  | 25 to 40 | 565 | 511 | 68 | 38.9 | 38.2 | 30.1 | 9,074 | 4,296 | 632 | 35.0 | 37.5 | 35.2 |
|  | 41 to 64 | 301 | 348 | 39 | 20.7 | 26.0 | 17.3 | 5,494 | 2,782 | 485 | 21.2 | 24.3 | 27.0 |
|  | Over 65 | 16 | 21 | 3 | 1.1 | 1.6 | 1.3 | 427 | 236 | 60 | 1.6 | 2.1 | 3.3 |
|  | Total | 1,452 | 1,338 | 226 | 100.0 | 100.0 | 100.0 | 25,895 | 11,467 | 1,797 | 100.0 | 100.0 | 100.0 |
|  |  | χ² p-value<0.05 | | |  |  |  | χ² p-value<0.01 | | |  |  |  |
| Symptoms | Asymptomatic | 107 | 33 | 7 | 7.4 | 2.5 | 3.1 | 327 | 290 | 48 | 1.3 | 2.5 | 2.7 |
|  | Symptomatic | 1,345 | 1,305 | 219 | 92.6 | 97.5 | 96.9 | 25,568 | 11,177 | 1,749 | 98.7 | 97.5 | 97.3 |
|  | Total | 1,452 | 1,338 | 226 | 100.0 | 100.0 | 100.0 | 25,895 | 11,467 | 1,797 | 100.0 | 100.0 | 100.0 |
|  |  | χ² p-value<0.01 | | |  |  |  | χ² p-value<0.01 | | |  |  |  |
| Occupation | Other | 310 | 300 | 80 | 21.3 | 22.4 | 35.4 | 741 | 5,050 | 4,015 | 41.2 | 19.5 | 35.0 |
|  | Agriculture | 338 | 187 | 23 | 23.3 | 14.0 | 10.2 | 426 | 12,398 | 3,320 | 23.7 | 47.9 | 29.0 |
|  | Domestic | 141 | 129 | 25 | 9.7 | 9.6 | 11.1 | 283 | 2,726 | 998 | 15.7 | 10.5 | 8.7 |
|  | Forestry | 39 | 20 | 4 | 2.7 | 1.5 | 1.8 | 30 | 409 | 170 | 1.7 | 1.6 | 1.5 |
|  | Hunter/fisherman | 264 | 102 | 38 | 18.2 | 7.6 | 16.8 | 17 | 95 | 82 | 0.9 | 0.4 | 0.7 |
|  | Miner | 191 | 495 | 38 | 13.2 | 37.0 | 16.8 | 50 | 576 | 704 | 2.8 | 2.2 | 6.1 |
|  | Tourist | 13 | 16 | 0 | 0.9 | 1.2 | 0.0 | 37 | 218 | 168 | 2.1 | 0.8 | 1.5 |
|  | Traveling | 47 | 34 | 5 | 3.2 | 2.5 | 2.2 | 112 | 479 | 562 | 6.2 | 1.8 | 4.9 |
|  | Road/dam builder | 9 | 12 | 4 | 0.6 | 0.9 | 1.8 | 26 | 40 | 370 | 1.4 | 0.2 | 3.2 |
|  | Missing | 100 | 43 | 9 | 6.9 | 3.2 | 4.0 | 75 | 3,904 | 1,078 | 4.2 | 15.1 | 9.4 |
|  | Total | 1,452 | 1,338 | 226 | 100.0 | 100.0 | 100.0 | 1,797 | 25,895 | 11,467 | 100.0 | 100.0 | 100.0 |
|  |  | χ² p-value<0.01 | | |  |  |  | χ² p-value<0.01 | | |  |  |  |
| Species | Mixed/Other | 27 | 22 | 17 | 1.9 | 1.6 | 7.5 | 584 | 137 | 20 | 2.3 | 1.2 | 1.1 |
|  | *P. falciparum* | 218 | 47 | 3 | 15.0 | 3.5 | 1.3 | 5,790 | 1,063 | 229 | 22.4 | 9.3 | 12.7 |
|  | *P. vivax* | 1,207 | 1,269 | 206 | 83.1 | 94.8 | 91.2 | 19,521 | 10,267 | 1,548 | 75.4 | 89.5 | 86.1 |
|  | Total | 1,452 | 1,338 | 226 | 100.0 | 100.0 | 100.0 | 25,895 | 11,467 | 1,797 | 100.0 | 100.0 | 100.0 |
|  |  | χ² p-value<0.01 | | |  |  |  | χ² p-value<0.01 | | |  |  |  |
| Schooling | Iliterate | 162 | 60 | 18 | 11.2 | 4.5 | 8.0 | 1656 | 574 | 66 | 6.4 | 5.0 | 3.7 |
|  | Elementary school (complete or incomplete) | 1,092 | 1,003 | 130 | 75.2 | 75.0 | 57.5 | 21,001 | 8,753 | 1,123 | 81.1 | 76.3 | 62.5 |
|  | High school (complete or incomplete) | 0 | 158 | 34 | 0.0 | 11.8 | 15.0 | 0 | 541 | 408 | 0.0 | 4.7 | 22.7 |
|  | College (complete or incomplete) | 39 | 27 | 11 | 2.7 | 2.0 | 4.9 | 601 | 467 | 75 | 2.3 | 4.1 | 4.2 |
|  | Not applicable | 103 | 71 | 28 | 7.1 | 5.3 | 12.4 | 1,306 | 437 | 66 | 5.0 | 3.8 | 3.7 |
|  | No info | 56 | 19 | 5 | 3.9 | 1.4 | 2.2 | 1,331 | 695 | 59 | 5.1 | 6.1 | 3.3 |
|  | Total | 1,452 | 1,338 | 226 | 100.0 | 100.0 | 100.0 | 25,895 | 11,467 | 1,797 | 100.0 | 100.0 | 100.0 |
|  |  | χ² p-value<0.01 | | |  |  |  | χ² p-value<0.01 | | |  |  |  |
| Distance from municipality of malaria occurrence to municipality of notification (in km) | Mean | 357.7 | 417.8 | 549.0 |  |  |  | 250.5 | 344.5 | 284.5 |  |  |  |
|  | Median | 276.8 | 276.8 | 545.4 |  |  |  | 159.4 | 245.0 | 197.6 |  |  |  |
|  | Min | 79.5 | 79.5 | 79.5 |  |  |  | 14.6 | 14.6 | 14.6 |  |  |  |
|  | Max | 1,431.5 | 1,513.9 | 1,414.6 |  |  |  | 2,303.3 | 2,476.8 | 2,270.1 |  |  |  |
|  | SD | 241.1 | 276.5 | 250.1 |  |  |  | 182.8 | 355.5 | 300.9 |  |  |  |
| Patient age (in years) | Mean | 28.9 | 31.5 | 25.8 |  |  |  | 29.1 | 31.0 | 32.7 |  |  |  |
|  | Median | 29.0 | 31.0 | 24.0 |  |  |  | 27.0 | 30.0 | 32.0 |  |  |  |
|  | Min | 0 | 0 | 0 |  |  |  | 0 | 0 | 0 |  |  |  |
|  | Max | 68 | 96 | 79 |  |  |  | 94 | 93 | 104 |  |  |  |
|  | SD | 15.3 | 15.0 | 15.4 |  |  |  | 15.3 | 15.4 | 16.5 |  |  |  |

χ² = Chi-Square test

SD: Standard Deviation
