# Supplementary material for: The impact of hydropower dam construction on malaria incidence: Space-time analysis in the Brazilian Amazon
Source: PLOS Glob Public Health. 2023 Mar 20;3(3):e0001683. doi: 10.1371/journal.pgph.0001683 (PMC10027221; doi:10.1371/journal.pgph.0001683)
Supplement: S3 Table — (DOCX) [file pgph.0001683.s005.docx]

**S3 Table.** Correlates of malaria infections whose sources were Altamira region (ATM) or Porto Velho municipality (PVH), 2004-2017 and 2007-2020 respectively

|  |  | ATM | | | | | | PVH | | | | | |
| --- | --- | --- | --- | --- | --- | --- | --- | --- | --- | --- | --- | --- | --- |
|  |  | Unadjusted model | | | Adjusted model | | | Unadjusted model | | | Adjusted model | | |
| Variable name |  | Odds ratio | (95% CI) | P-value | Odds ratio | (95% CI) | P-value | Odds ratio | (95% CI) | P-value | Odds ratio | (95% CI) | P-value |
| Intercept |  |  |  |  | 0.02 | (0.02-0.04) | <0.001 |  |  |  | 0.08 | (0.07-0.09) | <0.001 |
| Sex (Ref. Female) |  |  |  |  |  |  |  |  |  |  |  |  |  |
|  | Male | 1.78 | (1.63-1.94) | <0.001 | 1.30 | (1.17-1.45) | <0.001 | 1.5 | (1.47-1.54) | <0.001 | 1.21 | (1.18-1.25) | <0.001 |
| Age group (Ref. < 5 years old) |  |  |  |  |  |  |  |  |  |  |  |  |  |
|  | 5 to 15 | 1.17 | (0.97-1.41) | 0.112 | 1.01 | (0.77-1.31) | 0.964 | 0.74 | (0.69-0.78) | <0.001 | 1.01 | (0.93-1.08) | 0.889 |
|  | 16 to 24 | 2.67 | (2.23-2.20) | <0.001 | 1.85 | (1.39-2.48) | <0.001 | 1.32 | (1.25-1.40) | <0.001 | 1.84 | (1.7-1.99) | <0.001 |
|  | 25 to 40 | 4.12 | (3.48-4.91) | <0.001 | 2.65 | (1.99-3.53) | <0.001 | 1.52 | (1.44-1.61) | <0.001 | 2.11 | (1.95-2.28) | <0.001 |
|  | 41 to 64 | 4.41 | (3.69-5.29) | <0.001 | 3.02 | (2.26-4.04) | <0.001 | 1.5 | (1.42-1.58) | <0.001 | 1.99 | (1.84-2.15) | <0.001 |
|  | Over 65 | 2.05 | (1.41-5.90) | <0.001 | 1.81 | (1.16-2.76) | 0.007 | 1.27 | (1.16-1.40) | <0.001 | 1.85 | (1.66-2.06) | <0.001 |
| Symptoms (Ref. Asymptomatic) |  |  |  |  |  |  |  |  |  |  |  |  |  |
|  | Symptomatic | 0.73 | (0.61-0.87) | <0.001 | 0.60 | (0.5-0.73) | <0.001 | 0.92 | (0.85-1.00) | 0.068 | - | - | - |
| Occupation (Ref. Other) |  |  |  |  |  |  |  |  |  |  |  |  |  |
|  | Agriculture | 0.91 | (0.81-1.03) | 0.126 | 0.65 | (0.57-0.73) | <0.001 | 3.25 | (3.16-3.34) | <0.001 | 3.04 | (2.96-3.12) | <0.001 |
|  | Domestic | 0.97 | (0.84-1.12) | 0.718 | 0.95 | (0.81-1.12) | 0.056 | 1.01 | (0.97-1.05) | 0.598 | 1.11 | (1.06-1.15) | <0.001 |
|  | Forestry | 3.66 | (2.71-4.87) | <0.001 | 2.18 | (1.61-2.93) | <0.001 | 2.2 | (2.01-2.41) | <0.001 | 1.85 | (1.69-2.02) | <0.001 |
|  | Hunter/fisherman | 1.61 | (1.41-1.83) | <0.001 | 1.60 | (1.39-1.83) | <0.001 | 0.61 | (0.53-0.71) | <0.001 | 0.55 | (0.47-0.64) | <0.001 |
|  | Miner | 8.06 | (7.13-9.10) | <0.001 | 5.96 | (5.22-6.81) | <0.001 | 2.96 | (2.78-3.16) | <0.001 | 2.51 | (2.35-2.68) | <0.001 |
|  | Tourist | 1.75 | (1.15-2.56) | 0.005 | 1.46 | (0.95-2.14) | 0.068 | 0.67 | (0.60-0.73) | <0.001 | 0.65 | (0.59-0.72) | <0.001 |
|  | Traveling | 7.08 | (5.35-9.32) | <0.001 | 5.51 | (4.12-7.34) | <0.001 | 11.9 | (10.90-12.99) | <0.001 | 11.07 | (10.13-12.1) | <0.001 |
|  | Road/dam builder | 2.56 | (1.62-3.90) | <0.001 | 1.24 | (0.78-1.91) | 0.342 | 1.13 | (1.02-1.25) | 0.016 | 0.98 | (0.89-1.09) | 0.728 |
| Species (Ref. Mixed/Other) |  |  |  |  |  |  |  |  |  |  |  |  |  |
|  | *P. falciparum* | 1.7 | (1.30-2.27) | <0.001 | 1.22 | (0.92-1.65) | 0.182 | 0.51 | *(0.47-0.56)* | <0.001 | 0.53 | (0.48-0.58) | <0.001 |
|  | *P. vivax* | 1.85 | (1.45-2.40) | <0.001 | 1.48 | (1.15-1.94) | 0.003 | 0.41 | (0.38-0.45) | <0.001 | 0.47 | (0.43-0.51) | <0.001 |
| Schooling (Ref. Illiterate) |  |  |  |  |  |  |  |  |  |  |  |  |  |
|  | Elementary school (complete or incomplete) | 2.13 | (1.86-2.45) | <0.001 | 2.07 | (1.79-2.4) | <0.001 | 1.53 | (1.46-1.60) | <0.001 | 1.35 | (1.29-1.42) | <0.001 |
|  | High school (complete or incomplete) | 5.34 | (4.33-6.57) | <0.001 | 3.90 | (3.1-4.89) | <0.001 | 0.81 | (0.75-0.88) | <0.001 | 0.86 | (0.79-0.93) | <0.001 |
|  | College (complete or incomplete) | 2.57 | (2.57-1.94) | <0.001 | 2.27 | (1.7-3.02) | <0.001 | 1.28 | (1.19-1.38) | <0.001 | 1.21 | (1.12-1.31) | <0.001 |
