# Supplementary material for: The impact of hydropower dam construction on malaria incidence: Space-time analysis in the Brazilian Amazon
Source: PLOS Glob Public Health. 2023 Mar 20;3(3):e0001683. doi: 10.1371/journal.pgph.0001683 (PMC10027221; doi:10.1371/journal.pgph.0001683)
Supplement: S4 Table — (DOCX) [file pgph.0001683.s006.docx]

**S4 Table.** Correlates of malaria infections whose sources was Altamira region (ATM) – before (2007-2010), during (2011-2016) and after dam’s construction (2017-2020)

|  |  | Before | | | | | | During | | | | | | After | | | | | |
| --- | --- | --- | --- | --- | --- | --- | --- | --- | --- | --- | --- | --- | --- | --- | --- | --- | --- | --- | --- |
|  |  | Unadjusted model | | | Adjusted model | | | Unadjusted model | | | Adjusted model | | | Unadjusted model | | | Adjusted model | | |
| Variable name |  | Odds ratio | (95% CI) | P-value | Odds ratio | (95% CI) | P-value | Odds ratio | (95% CI) | P-value | Odds ratio | (95% CI) | P-value | Odds ratio | (95% CI) | P-value | Odds ratio | (95% CI) | P-value |
| Intercept |  | - | - | - | 0.14 | (0.06-0.14) | <0.001 | - | - | - | 0.01 | (0-0.01) | <0.001 | - | - | - | 1.37 | (0.09-1.37) | 0.127 |
| Sex (Ref. Female) |  |  |  |  |  |  |  |  |  |  |  |  |  |  |  |  |  |  |  |
|  | Male | 1.87 | (1.65-2.13) | <0.001 | 1.59 | (1.16-1.59) | <0.001 | 1.71 | (1.50-1.96) | <0.001 | 1.21 | (1.02-1.45) | 0.029 | 1.82 | (1.35-2.47) | <0.001 | 2.57 | (1.29-2.57) | <0.001 |
| Age group (Ref. < 5 years) |  |  |  |  |  |  |  |  |  |  |  |  |  |  |  |  |  |  |  |
|  | 5 to 15 | 1.17 | (0.91-1.52) | 0.211 | 1.12 | (0.56-1.12) | 1.187 | 0.09 | (1.67-0.54) | 0.586 | 1.22 | (0.78-1.91) | 0.387 | 1.31 | (0.72-2.52) | 0.391 | 4.22 | (0.87-4.22) | 0.105 |
|  | 16 to 24 | 2.05 | (1.61-2.63) | <0.001 | 1.56 | (0.73-1.56) | 0.759 | 1.33 | (0.16-8.57) | <0.001 | 3.24 | (1.98-5.33) | <0.001 | 2.61 | (1.45-4.99) | 0.002 | 10.08 | (1.62-10.08) | 0.002 |
|  | 25 to 40 | 3.57 | (2.85-4.51) | <0.001 | 2.6 | (1.23-2.6) | 0.002 | 1.67 | (0.15-11.18) | <0.001 | 3.8 | (2.34-6.21) | <0.001 | 3.27 | (1.84-6.20) | <0.001 | 11.23 | (1.81-11.23) | 0.001 |
|  | 41 to 64 | 3.41 | (2.68-4.37) | <0.001 | 2.68 | (1.24-2.68) | 0.002 | 1.82 | (0.15-11.9) | <0.001 | 4.25 | (2.59-6.98) | <0.001 | 4.37 | (2.33-8.65) | <0.001 | 20.28 | (3.03-20.28) | <0.001 |
|  | Over 65 | 1.45 | (0.81-2.43) | 0.187 | 1.72 | (0.49-1.72) | 0.856 | 1.94 | (0.27-4.40) | <0.001 | 2.74 | (1.35-5.44) | 0.004 | 1.28 | (0.28-4.20) | 0.704 | 11.81 | (0.55-11.81) | 0.166 |
| Symptoms (Ref. Asymptomatic) |  |  |  |  |  |  |  |  |  |  |  |  |  |  |  |  |  |  |  |
|  | Symptomatic | 0.42 | (0.34-0.53) | <0.001 | 0.41 | (0.25-0.41) | <0.001 | 1.44 | (1.03-2.12) | 0.043 | - | - | - | 2.74 | (1.35-6.56) | 0.01 | - | - | - |
| Occupation (Ref. Other) |  |  |  |  |  |  |  |  |  |  |  |  |  |  |  |  |  |  |  |
|  | Agriculture | 1.01 | (0.86-1.19) | 0.902 | 0.87 | (0.62-0.87) | <0.001 | 0.8 | (0.66-0.96) | 0.22 | 0.53 | (0.43-0.65) | <0.001 | 1.2 | (0.71-1.94) | 0.472 | 1.63 | (0.54-1.63) | 0.865 |
|  | Domestic | 0.75 | (0.61-0.92) | 0.005 | 0.96 | (0.59-0.96) | 0.02 | 1.35 | (1.08-1.67) | 0.005 | 1.25 | (0.97-1.6) | 0.077 | 1.27 | (0.77-2.04) | 0.32 | 2.59 | (0.9-2.59) | 0.108 |
|  | Forestry | 2.82 | (1.93-4.03) | <0.001 | 2.57 | (1.21-2.57) | 0.003 | 6.45 | (3.67-10.96) | <0.001 | 3.33 | (1.88-5.73) | <0.001 | 7.46 | (1.73-3.21) | 0.005 | 22.45 | (1.09-22.45) | 0.032 |
|  | Hunter/fisherman | 1.7 | (1.43-2.02) | <0.001 | 2.16 | (1.5-2.16) | <0.001 | 1.48 | (1.16-1.86) | 0.001 | 1.43 | (1.11-1.83) | 0.004 | 1.01 | (0.66-1.51) | 0.951 | 1.49 | (0.6-1.49) | 0.836 |
|  | Miner | 2.81 | (2.31-3.41) | <0.001 | 2.9 | (1.91-2.9) | <0.001 | 34.34 | (28.12-42.10) | <0.001 | 19.71 | (15.93-24.48) | <0.001 | 3.01 | (1.92-4.67) | <0.001 | 3.24 | (1.18-3.24) | 0.009 |
|  | Tourist | 1.08 | (0.58-1.85) | 0.8 | 1.62 | (0.5-1.62) | 0.823 | 4.62 | (2.51-8.06) | <0.001 | 3.21 | (1.72-5.69) | <0.001 | - | - | - | - | - |  |
|  | Traveling | 7.38 | (5.00-10.78) | <0.001 | 8.5 | (3.84-8.5) | <0.001 | 7.73 | (4.95-11.88) | <0.001 | 5.79 | (3.64-9.09) | <0.001 | 3.1 | (0.96-8.62) | 0.037 | 10.87 | (1-10.87) | 0.035 |
|  | Road/dam builder | 6.23 | (2.64-13.79) | <0.001 | 7.33 | (1.37-7.33) | 0.004 | 1.79 | (0.92-3.17) | 0.153 | 0.87 | (0.45-1.57) | 0.672 | 3.31 | (0.88-1.04) | 0.05 | 4.07 | (0.28-4.07) | 0.8184 |
| Species (Ref. Mixed/Other) |  |  |  |  |  |  |  |  |  |  |  |  |  |  |  |  |  |  |  |
|  | P. falciparum | 0.8 | (0.53-1.24) | 0.295 | - | - | - | 5.12 | (3.07-8.77) | <0.001 | 2.99 | (1.71-5.36) | <0.001 | 0.23 | (0.04-1.12) | 0.08 | 0.42 | (0.01-0.42) | 0.004 |
|  | *P. vivax* | 0.74 | (0.50-1.13) | 0.137 | - | - | - | 5.45 | (3.64-8.60) | <0.001 | 3.61 | (2.36-5.83) | <0.001 | 0.06 | (0.02-0.15) | <0.001 | 0.09 | (0.01-0.09) | <0.001 |
| Schooling (Ref. Illiterate) |  |  |  |  |  |  |  |  |  |  |  |  |  |  |  |  |  |  |  |
|  | Elementary school (complete or incomplete) | 2.34 | (1.98-2.78) | <0.001 | 2.86 | (1.99-2.86) | <0.001 | 1.41 | (1.08-1.87) | 0.013 | 1.11 | (0.83-1.52) | 0.491 | 2.1 | (1.29-3.64) | 0.004 | 3.35 | (1.11-3.35) | 0.024 |
|  | High school (complete or incomplete) | - | - | - | - | - | - | 3.36 | (2.45-4.66) | <0.001 | 1.82 | (1.27-2.65) | 0.001 | 4.41 | (2.40-8.34) | <0.001 | 7.16 | (1.88-7.16) | <0.001 |
|  | College (complete or incomplete) | 1.87 | (1.28-2.66) | <0.001 | 2.67 | (1.25-2.67) | 0.002 | 3.51 | (2.08-5.81) | <0.001 | 2.17 | (1.22-3.81) | 0.007 | 9.99 | (3.93-25.36) | <0.001 | 23.14 | (3.09-23.14) | <0.001 |
