# Supplementary material for: The impact of hydropower dam construction on malaria incidence: Space-time analysis in the Brazilian Amazon
Source: PLOS Glob Public Health. 2023 Mar 20;3(3):e0001683. doi: 10.1371/journal.pgph.0001683 (PMC10027221; doi:10.1371/journal.pgph.0001683)
Supplement: S5 Table — (DOCX) [file pgph.0001683.s007.docx]

**S5 Table.** Correlates of malaria infections whose sources was Porto Velho municipality (PVH) – before (2004-2007), during (2008-2013) and after dam’s construction (2014-2017)

|  |  | Before | | | | | | During | | | | | | After | | | | | |
| --- | --- | --- | --- | --- | --- | --- | --- | --- | --- | --- | --- | --- | --- | --- | --- | --- | --- | --- | --- |
|  |  | Unadjusted model | | | Adjusted model | | | Unadjusted model | | | Adjusted model | | | Unadjusted model | | | Adjusted model | | |
| Variable name |  | Odds ratio | (95% CI) | P-value | Odds ratio | (95% CI) | P-value | Odds ratio | (95% CI) | P-value | Odds ratio | (95% CI) | P-value | Odds ratio | (95% CI) | P-value | Odds ratio | (95% CI) | P-value |
| Intercept |  |  |  |  | 0.04 | (0.03-0.05) | <0.001 |  |  |  | 0.21 | (0.15-0.27) | <0.001 |  |  |  | 0.13 | (0.08-0.2) | <0.001 |
| Sex (Ref. Female) |  |  |  |  |  |  |  |  |  |  |  |  |  |  |  |  |  |  |  |
|  | Male | 1.52 | (1.48-1.57) | <0.001 | 1.4 | (1.36-1.45) | <0.001 | 1.45 | (1.39-1.51) | <0.001 | 1.14 | (1.09-1.19) | <0.001 | 1.41 | (1.27-1.58) | <0.001 | 1.11 | (0.99-1.25) | 0.068 |
| Age group (Ref. < 5 years) |  |  |  |  |  |  |  |  |  |  |  |  |  |  |  |  |  |  |  |
|  | 5 to 15 | 0.79 | (0.74-0.85) | <0.001 | 0.96 | (0.88-1.05) | 0.408 | 0.62 | (0.56-0.70) | <0.001 | 1.02 | (0.88-1.18) | 0.804 | 0.7 | (0.51-0.99) | 0.41 | - | - | - |
|  | 16 to 24 | 1.44 | (1.34-1.53) | <0.001 | 1.61 | (1.46-1.76) | <0.001 | 1.22 | (1.10-1.35) | <0.001 | 2.08 | (1.79-2.42) | <0.001 | 1.08 | (0.79-1.50) | 0.629 | - | - | - |
|  | 25 to 40 | 1.73 | (1.62-1.85) | <0.001 | 1.91 | (1.74-2.1) | <0.001 | 1.4 | (1.27-1.56) | <0.001 | 2.33 | (2.02-2.7) | <0.001 | 1.12 | (0.82-1.55) | 0.468 | - | - | - |
|  | 41 to 64 | 1.71 | (1.60-1.83) | <0.001 | 1.86 | (1.69-2.04) | <0.001 | 1.4 | (1.27-1.56) | <0.001 | 2.17 | (1.87-2.52) | <0.001 | 1.13 | (0.83-1.57) | 0.429 | - | - | - |
|  | Over 65 | 1.42 | (1.25-1.60) | <0.001 | 1.68 | (1.46-1.93) | <0.001 | 1.28 | (1.08-1.51) | 0.003 | 2.23 | (1.83-2.71) | <0.001 | 1.01 | (0.67-1.51) | 0.956 | - | - | - |
| Symptoms (Ref. Asymptomatic) |  |  |  |  |  |  |  |  |  |  |  |  |  |  |  |  |  |  |  |
|  | Symptomatic | 1.58 | (1.41-1.78) | <0.001 | 1.74 | (1.55-1.96) | <0.001 | 0.41 | (0.36-0.47) | <0.001 | 0.44 | (0.38-0.51) | <0.001 | 0.49 | (0.35-0.68) | <0.001 | 0.59 | (0.42-0.84) | 0.002 |
| Occupation (Ref. Other) |  |  |  |  |  |  |  |  |  |  |  |  |  |  |  |  |  |  |  |
|  | Agriculture | 4.08 | (3.93-4.22) | <0.001 | 3.78 | (3.65-3.92) | <0.001 | 1.97 | (1.88-2.07) | <0.001 | 1.89 | (1.8-1.99) | <0.001 | 2.64 | (2.30-3.01) | <0.001 | 2.57 | (2.24-2.95) | <0.001 |
|  | Domestic | 2.17 | (2.06-2.28) | <0.001 | 2.54 | (2.41-2.69) | <0.001 | 0.44 | (0.41-0.48) | <0.001 | 0.47 | (0.44-0.51) | <0.001 | 0.44 | (0.38-0.51) | <0.001 | 0.44 | (0.38-0.51) | <0.001 |
|  | Forestry | 2.81 | (2.51-3.14) | <0.001 | 2.3 | (2.05-2.57) | <0.001 | 1.4 | (1.18-1.65) | <0.001 | 1.2 | (1.01-1.41) | 0.031 | 4.01 | (2.53-6.22) | <0.001 | 3.79 | (2.39-5.9) | <0.001 |
|  | Hunter/fisherman | 0.78 | (0.63-0.96) | 0.026 | 0.67 | (0.54-0.82) | <0.001 | 0.48 | (0.38-0.60) | <0.001 | 0.45 | (0.35-0.56) | <0.001 | 0.52 | (0.31-0.84) | 0.012 | 0.52 | (0.3-0.83) | 0.01 |
|  | Miner | 2.6 | (2.36-2.86) | <0.001 | 2.21 | (2-2.43) | <0.001 | 3.35 | (3.05-3.67) | <0.001 | 2.96 | (2.69-3.25) | <0.001 | 3.2 | (2.26-4.46) | <0.001 | 3.09 | (2.18-4.31) | <0.001 |
|  | Tourist | 1.67 | (1.44-1.93) | <0.001 | 1.64 | (1.41-1.89) | <0.001 | 0.36 | (0.31-0.42) | <0.001 | 0.35 | (0.3-0.41) | <0.001 | 0.52 | (0.36-0.72) | <0.001 | 0.53 | (0.37-0.74) | <0.001 |
|  | Traveling | 10.3 | (9.05-11.72) | <0.001 | 9.24 | (8.11-10.54) | <0.001 | 13.12 | (11.50-14.97) | <0.001 | 12.46 | (10.91-14.24) | <0.001 | 13.79 | (10.07-19.04) | <0.001 | 13.28 | (9.67-18.38) | <0.001 |
|  | Road/dam builder | 4.23 | (2.90-6.06) | <0.001 | 3.29 | (2.25-4.72) | <0.001 | 0.97 | (0.87-1.09) | 0.701 | 0.83 | (0.74-0.93) | 0.001 | 1.58 | (1.01-2.39) | 0.034 | 1.56 | (0.99-2.35) | 0.044 |
| Species (Ref. Mixed/Other) |  |  |  |  |  |  |  |  |  |  |  |  |  |  |  |  |  |  |  |
|  | P. falciparum | 0.53 | (0.48-0.59) | <0.001 | 0.54 | (0.49-0.6) | <0.001 | 0.41 | (0.33-0.50) | <0.001 | 0.41 | (0.33-0.51) | <0.001 | 0.58 | (0.36-0.98) | 0.032 | - | - | - |
|  | *P. vivax* | 0.49 | (0.44-0.54) | <0.001 | 0.51 | (0.46-0.57) | <0.001 | 0.33 | (0.27-0.41) | <0.001 | 0.41 | (0.34-0.51) | <0.001 | 0.71 | (0.45-1.18) | 0.17 | - | - | - |
| Schooling (Ref. Illiterate) |  |  |  |  |  |  |  |  |  |  |  |  |  |  |  |  |  |  |  |
|  | Elementary school (complete or incomplete) | 1.57 | (1.49-1.66) | <0.001 | 1.4 | (1.32-1.48) | <0.001 | 1.52 | (1.40-1.67) | <0.001 | 1.33 | (1.21-1.46) | <0.001 | 1.74 | (1.35-2.27) | <0.001 | 1.63 | (1.26-2.15) | <0.001 |
|  | High school (complete or incomplete) | - | - | - | - | - | - | 1.07 | (0.95-1.21) | 0.241 | 1.04 | (0.91-1.19) | 0.559 | 1.29 | (0.99-1.71) | 0.06 | 1.34 | (1.02-1.78) | 0.038 |
|  | College (complete or incomplete) | 1.17 | (1.06-1.30) | 0.001 | 1.17 | (1.05-1.3) | 0.004 | 1.64 | (1.44-1.87) | <0.001 | 1.41 | (1.23-1.61) | <0.001 | 1.85 | (1.31-2.64) | <0.001 | 1.88 | (1.31-2.7) | <0.001 |
